# Potential of rare actinomycetes in the production of metabolites against multiple oxidant agents

**DOI:** 10.1080/13880209.2017.1417451

**Published:** 2017-12-23

**Authors:** Fatemeh Mohammadipanah, Mana Momenilandi

**Affiliations:** a Microbial Biotechnology Laboratory, Department of Microbiology, School of Biology and Center of Excellence in Phylogeny of Living Organisms, College of Science, University of Tehran, Tehran, Iran;; b UTMC-University of Tehran Microorganisms Collection, University of Tehran, Tehran, Iran

**Keywords:** Secondary metabolites, natural antioxidants, radical scavenging agents, enzyme inhibition

## Abstract

**Context:** Actinobacteria are a precious source of novel bioactive metabolites with potential pharmaceutical applications.

**Objectives:** Representatives of 11 genera of rare Actinobacteria were selected for the evaluation of antioxidant activity.

**Material and methods:** Fermentation broths of the Actinobacteria were extracted and dosage of 10 to 2000 µg/mL were applied for *in vitro* antioxidant-related bioassays. Cytotoxicity was assessed at the concentration of 2.5–20 µg/mL.

**Results:** In the DPPH scavenging activity, 15 out of 52 extracts showed 17.0–26.8% activity in quantitative evaluation. Metabolites of five prominent antioxidant producing strains protected the DNA (pUC19) against UV-induced photolyzed H_2_O_2_-oxidative degradation. The potent antioxidant extracts inhibited two oxidative enzymes of xanthine oxidase in the range of 17.5–45.2% (three extracts had IC_50_ less than allopurinol) and lipoxygenase in the range of 36–55% (all five extracts had IC_50_ values less than daidzein). All these extracts could also protect eythrocytes from iron-induced hemolysis with ED_50_ values in a range of 0.014–1.25 mg/mL. Growth restoration of the yeast cells lacking the sod1 gene was observed by the antioxidant metabolite of *Saccharothrix ecbatanensis* UTMC 537 at the concentration of 1 mg/mL.

**Conclusions:** The presence of nonidentical metabolites might be responsible for antioxidant and enzyme inhibitory activities of *S. ecbatanensis*, newly described actinobacterium in family *Pseudonocardiaceae*. The scavenging of the free electrons, protection of DNA and model yeast cells against oxidative stress, in addition to the inhibition of the oxidating enzymes are the main mechanisms of the antioxidant effect of the introduced resource in this study.

## Introduction

Oxidative stress represents an imbalance between the production of reactive oxygen species (ROS) or oxygen radicals and the biological system’s ability to inhibit the reactive oxygen intermediates (ROI) or precursors of them, to alleviate the resulting damage (Baynes 1991). Oxidative stress plays an important role in the development of many diseases, such as Alzheimer’s, Parkinson, rheumatoid arthritis, cancer, and cardiovascular diseases (Halliwell 1996, 2006) and are involved in a variety of pathological implications, as it can be damaging to any type of macromolecules including DNA, proteins and lipids (Halliwell 1991).

Antioxidants can react with free radicals, scavenge them and inhibit oxidation created under physiological conditions, thereby function in prevention and treatment of the related diseases (Niki 2010). In addition to the medicinal purposes, antioxidants have numerous other applications, including in the cosmetic or food industry as lipid oxidation preventing agents (Wang and Shahidi 2014). In order to reduce the ROS-induced oxidative damage, both synthetic and natural antioxidants are used. Although synthetic antioxidants are strong radical scavengers, they usually have side effects, as the implication of *tert*‐butylhydroquinone (TBHQ) in cancer has previously been reported (Hirose et al. 1993). As a result, in order to protect against free radicals and retard the progression of many chronic diseases, the discovery of natural nontoxic antioxidants is demanded. In the investigation of natural antioxidants, a number of compounds have been obtained from different sources, mainly from plants, like polyphenols and phytosterols (Lu and Foo 2001; Tan and Shahidi 2012). However, the largest structural diversity of natural compounds belongs to microorganism-derived molecules. Among the bacterial biologically active compounds, almost 45% are produced by Gram-positive, often Actinobacteria, that are known as remarkable producers of secondary metabolites. A variety of actinobacterial antioxidants such as dihydroherbimycin A (Chang and Kim 2007), *N*-carbamoyl-2,3-dihydroxybenzamide, 2-acetamido-3-(2,3-dihydroxybenzoylthio) propanoic acid (Sugiyama and Hirota 2009), 2-allyloxyphenol (Arumugam et al. 2010), and phenazines (Abdel-Mageed et al. 2010) and Saccharomonopyrone A (Yim et al. 2017) have been identified to date. Among Actinobacteria, the majority of the biologically active compounds have been discovered from the genus *Streptomyces* (Berdy 2005; Zotchev 2012). Due to the high possibility of finding already known antioxidant metabolites, less abundant or culturable strains of Actinobacteria such as rare genera should be targeted for the discovery of new bioactive compounds (Subramani and Aalbersberg 2013).

In order to reduce the re-isolation of known antioxidant compounds from the *Streptomyces* genus, genera other than the large group of *Streptomyces* were analyzed in this study. The current work presents an attempt to determine the potential of producing metabolites with antioxidant properties in rare genera of Actinobacteria, which have multiple functional mechanisms of antioxidant activity.

## Materials and methods

### Chemicals

1,1-Diphenyl-2-picrylhydrazyl (DPPH), NADH (nicotinamide adenine dinucleotide), lipoxygenase, linoleic acid, xanthine (99%), xanthine oxidase (25 units), BHT (butylated hydroxytoluene), PMS (phenazinemethosulfate), glutathione and ascorbic acid were purchased from Sigma-Aldrich (St. Louis, MO, USA) and silica gel TLC 60 F254 from Merck (Darmstadt, Germany).

### Actinobacterial source of antioxidant compounds

Actinobacterial strains (52 strains) from 11 genera of *Nonomuraea* sp., *Micromonospora* sp., *Saccarothrix* sp., *Nocardiopsis* sp., *Amycolatopsis* sp., *Kribbella* sp., *Nocardia* sp., *Actinophytocola* sp., *Streptosporangium* sp., *Promicromonospora* sp. and *Actinokineospora* sp. were obtained from the University of Tehran Microorganisms Collection (UTMC). All of the strains were preserved in the vapor of liquid nitrogen and as glycerol suspensions (30% W/V) at −70 °C.

### Metabolite production and extraction

The isolates were grown in ISP2 broth medium (PH 7.4) in a shaker incubator (220 rpm) for 2–3 days at 28 °C. After sufficient growth of bacteria, 5% of the seeding medium was inoculated into a new ISP2 broth as the fermentation medium and incubated for 7–9 days at 28 °C with 220 rpm.

In order to extract the produced extracellular metabolite, fermentation broths were centrifuged for 10 min at 4000 rpm. The supernatants were extracted with an equal volume of ethyl acetate with vigorous shaking up to 60 min for two successive times. Then the ethyl acetate fractions were concentrated at 35 °C. After drying the extract concentrate, the extracts were stored at −20 °C and −70 °C for further experiments.

### Antioxidant activity assays

#### Rapid TLC screening by 1,1-diphenyl-2-picrylhydrazyl (DPPH) radical scavenging assay

The crude extracts were developed on TLC silica gel 60 F254 plates in methanol/dichloromethane (10:90) mixture. Radical scavenging activity of all extracts was recognized by the reduction of color intensity following spraying with a 0.05% DPPH/methanol solution. After 30 min incubation in a dark place, the formation of yellow spots against a purple background (similar to ascorbic acid and BHT as positive controls) indicated the presence of the antioxidant activity (Braca et al. 2002) while extract of non-inoculated fermentation medium was used as the negative control.

#### DPPH radical scavenging activity assay

The antioxidant activity of the crude extracts was measured according to the method of Arumugam et al. (2010) in a 96-well microtitre plate format. The concentration of 10 μg/mL of the extracts was added to 190 μL of a 100 μM DPPH solution in methanol. Following incubation at 37 °C for 30 min in darkness, the absorbance of each solution was determined at 492 nm using a Hyperion microplate reader system (Miami, FL, USA) ascorbic acid and BHT were used as positive controls in a similar concentration to the extracts (10 μg/mL). Extract of non-inoculated fermentation medium was used as a negative control and wells without adding extracts were considered as blank samples. Triplicate measurements were made and the ability to scavenge the DPPH radical was calculated by the following Formula 1, where A_0_ is the absorbance of the control and A_1_ is the absorbance of the sample.

(1) $$\text{Radical scavenging activity }\left(\%\right)=\left[\left({\text{A}}_{0}–{\text{ A}}_{\text{1}}/{\text{A}}_{0}\right)\times \text{1}00\right]$$

### Toxicity evaluation using brine shrimp

The eggs of *Artemia salina* were hatched in artificial seawater under a light source and aeration at 27–30 °C (Kester et al. 1967). After 72 h, hatched eggs were collected from the bright side of the hatching container and active nauplii were used for the bioassay (Olaleye 2007).

The extracts (at final concentrations of 2.5, 5, and 10 μg/mL) were dissolved in DMSO (final concentration <5 mM) and diluted with artificial sea water in a 48-well microtitre plate. DMSO was used as a negative control at the same concentration and potassium dichromate (0.5 mM) was used as the positive control. About 10–15 larvae were placed in each well using a micropipette and incubated at room temperature for 24 h. After 6, 12, 18, and 24 h, the number of dead nauplii in each plate was counted. The percentage of lethality was determined by comparison of surviving larvae in the test and control wells.

#### Superoxide radical scavenging activity

The scavenging ability of the Actinobacterial extracts was evaluated by the generation of superoxide anion (O_2_^−^) in a PMS-NADH system, which was determined by the reduction of NBT (Liu et al. 1997). Superoxide radicals were generated in 1 mL of 20 mM Tris–HCl buffer pH 8.0 containing 0.078 mM NADH, 0.05 mM nitrobluetetrazolium (NBT), 0.01 mM phenazinemethosulphate (PMS), and methanol (40 μg/mL). The color reaction of the superoxide radicals and NBT were measured at 545 nm. BHT (40 μg/mL) was used as a positive control. Triplicate measurements were made and results were expressed as a percentage of inhibition of superoxide radicals according to Formula 1.

### Xanthine oxidase inhibitory activity

The xanthine oxidase inhibitory activity of the extracts was measured spectrophotometrically at 295 nm (Sweeney et al. 2001). The assay mixture consisted of 400 μL PBS (pH 8) and 20 μL enzyme solution (0.5 unit/mL) in the same buffer, 250 μL of Actinobacterial extract (in a final concentration of 12.25 μg/mL) dissolved in DMSO (in a final concentration of <1 mM) and phosphate buffer saline. This mixture was preincubated for 20 min at 37 °C, and then the reactions were initiated by adding 350 μL of 0.15 mM xanthine solution. Then the assay mixture was incubated at 37 °C for 20 min and the absorbance was measured using a PerkinElmer LAMBDA 25 UV/Vis Spectrophotometer (Hopkinton, MA, USA). Allopurinol was used as the positive control at the same concentration. The blank sample contained all test solutions except the extracts. XO inhibitory activity was expressed as the inhibition percentage of XO by Formula 1.

### Lipoxygenase inhibitory activity

Extracts were dissolved in DMSO and diluted with borate buffer. The reaction mixture consisted of Actinobacterial extracts at the final concentration of 4 μg/mL, 10 μL enzyme solution prepared from 500 units/mL stock in 0.2 M borate buffer (PH 9) and linoleic acid solution (0.134 mM) as the substrate. The stock solution of linoleic acid was prepared using Tween-20 and sodium borate buffer at pH 9.0 and then the total Tween-20 content was adjusted to below 0.002% in the final volume of the assay. The reaction mixture was incubated for 15 min at room temperature and then was kept for 5 min in a boiling water bath in order to terminate the reaction. Lipoxygenase inhibitory activity of all extracts was measured at 234 nm (Kubo et al. 2006). Daidzein was employed as the positive control at the same concentration of extracts and the solution containing 0.3 mM DMSO was used as the blank sample and reaction mixtures without the addition of enzyme was used as the negative control.

### DNA damage inhibition assay

The potential of extracts to prevent DNA damage was determined by photolyzing the pUC19 plasmid DNA (Tharakan et al. 2005). One aliquot of pUC19 (2 µL) was placed into the polyethylene microcentrifuge tube and then 4 µL of each extract (1 and 2 mg/mL) was separately added to the tubes. Before irradiating, 3 µL of 3% H_2_O_2_ was added and the samples were placed directly on the surface of a UV transilluminator (300 nm) for 5 min at room temperature. After incubation, each sample was mixed with 1 µL of loading dye and was analyzed by electrophoresis on agarose gel (1%). The gel was stained with ethidium bromide and DNA bands of the samples were analyzed using a gel doc system against untreated pUC19 with extracts.

### Anti-hemolytic activity

The antioxidant activity of the extracts was measured based on the inhibition of erythrocyte hemolysis, as described by Thephinlap et al. (2013). Blood was obtained by venipuncture from a healthy male volunteer and collected in heparinized tubes. Erythrocytes separated from the plasma and the buffy coat was washed three times with 10 mM phosphate buffer saline (PBS) at pH 7.4 and centrifuged at 2500 rpm for 5 min. During the last washing, the erythrocytes were obtained by centrifugation at 2500 rpm for 10 min and the packed cells were resuspended in 10 volumes of PBS with pH 7.4 and applied for the assay.

RBC suspension was incubated with 2.5 mM ferrous sulfate and 100 μg/mL of the extracts at 37 °C for 30 min. After incubation, 4 mL of PBS was added and further centrifuged at 2500 rpm for 5 min. Hemolysis was determined by measuring the OD of the supernatant at 540 nm. The reaction without extract and glutathione was used as the blank and positive control, respectively. Samples without the addition of ferrous sulfate and hemolysis induction were considered as negative control. The percentage of anti-hemolysis was calculated using Formula 1.

### Determination of cytotoxicity using MTT

Human umbilical vein endothelial cells (HUVEC) were obtained from the Pasteur Institute of Iran. They were cultured in Dulbecco modified essential medium (DMEM) in a humidified atmosphere of 5% CO_2_ at 37 °C until confluency was obtained. Cell lines in the exponential growth phase were washed, trypsinized, and resuspended in culture medium. Cells were plated at 1 × 10^4^ cells per well in a 96-well microtitre plate and incubated for 24 h during which a partial monolayer formed. The cells were exposed to concentrations of 5, 10, and 20 μg/mL of extracts dissolved in DMSO and were incubated at 37 °C in a humidified incubator with 5% CO_2_ for a period of 24 h. The cells treated with the solvent DMSO without extract were considered as negative control and cells treated with no additive were considered as blank wells. At the end of 24 h, cell viability was determined by MTT reagent (Mosmann 1983).

### Assay of yeast growth restoration by antioxidants

The potential of the most potent extract with antioxidant activity in the protection of yeast cells against induced oxidant stress was evaluated (Koziol et al. 2005). The wild-type SP-4 (MATα leu1 arg4) of *Saccharomyces cerevisiae* and its CuZnSOD disruptant *sod1::natMX* were obtained from Prof. S. Bednarska from University of Rzeszow. Dilutions (1 × 10^4^, 1 × 10^3^, 1 × 10^2^, 10 cells/mL) of yeast exponential phase cultures in a volume of 10 µL were inoculated on solid YPD medium (Sigma, Y-1375), with or without an addition of 0.8 M NaCl and antioxidant extract. Stock solutions of extract and ascorbic acid (as a positive control) were added to sterile media at a final concentration of 1 mg/mL. The media without addition of extract and with an addition of the extract from uninoculated fermentation medium were considered as blank and negative control, respectively. Media were cooled to just above the solidification point before addition of antioxidants. Plates were incubated at 28 °C and examined after 72 h.

### Statistical analysis

All assays were performed in triplicate. Data were expressed as a mean ± standard deviation for each variable. Statistical comparisons using one way analysis of variance (ANOVA) was considered for comparison of bioassay results between samples and standard compounds and values of *p* < 0.05 was regarded as significant.

### Metabolite analysis of the most active strain usingUPLC-HRMS

The crude extract of the *S. ecbatanensis* was subjected to mass analysis and HRESIMS data were recorded on a maXis UHR-TOF mass spectrometer (Bruker, Billerica, MA, USA). Analytical RP HPLC was carried out with an Agilent 1200 (Santa Clara, CA, USA) HPLC system equipped with a diodearray UV detector (DAD) at 200–600 nm connected with the maXis ESI TOF mass spectrometer. HPLC conditions: Waters Acquity UPLC BEH C18 column 50 × 2.1 mm, 1.7 μm, solvent A: H2O, 0.1% formic acid; solvent B: acetonitrile, 0.1% formic acid; gradient system: 5% B for 1 min, increasing to 95% B in 20 min; flow rate 0.6 mL/min; column oven temperature 40 °C. The Data Analysis software (Compass-software, Bruker, USA) was used for analysis of the spectra and calculation of molecular formula including the isotopic pattern (Smart Formula algorithm). New compounds were detected by comparison of molecular weights and UV spectra with the characteristics of the known natural derived compounds in the databases of (Dictionary of Natural Products, CRC press) and SciFinder (Chemical Abstract Service, USA).

## Results and discussion

### DPPH radical scavenging activity

**Figure 1.**
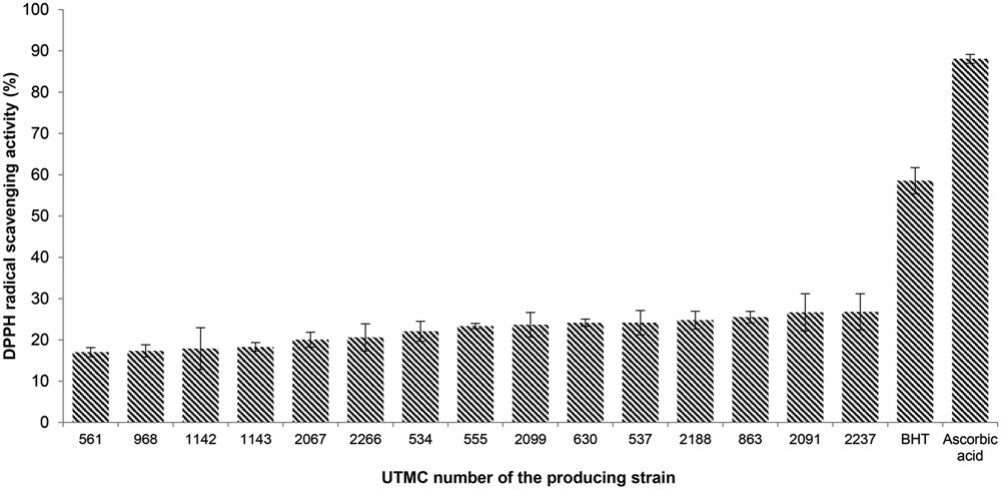
DPPH radical scavenging activity of extracts and positive controls at the concentration of 10 μg/mL. Data are mean ± SD of three experiments.

More than 30 out of 52 Actinobacterial extracts exhibited antioxidant properties in the TLC assay. According to the intensity of yellow spots against a purple background or the frequency of yellow spots in an identical extract, 15 extracts were selected for quantitative DPPH assay. As is presented in Figure 1, DPPH scavenging activity was observed in the range of 17.0–26.8%, while BHT and ascorbic acid showed 58.6% and 88.1% activity in similar concentrations, respectively. The extract with the highest activity belonged to *Nonomuraea* sp. UTMC 2237.

Passari et al. (2017) have recently reported that the effective DPPH radical scavenging activity in the extract of four endophytic actinobacteria (all from *Streptomyces* genus) with the maximum scavenging activity (IC_50_ value of 43.2 μg/mL). Another endophytic *Streptomyces* sp. described by Christhudas et al. (2013) have shown scavenging activity of DPPH radical (IC_50_ 435.31 μg/mL), and superoxide anion radical (IC_50_, 220.31 μg/mL).

In another recent study, a mangrove-derived *Streptomyces* sp. exhibited radicals reduction 5.80% to 22.03% of DPPH at doses ranging from 0.5 to 4 mg/mL (Tan LT-H et al. 2017). The extract of marine *Streptomyces* sp. has also been reported with 43.2% DPPH radical scavenging activity at a high concentration of 10 mg/mL (Thenmozhi and Kannabiran 2012). While another marine *Streptomyces* has reported for scavenging activity of DPPH radical with the IC_50_, 41.09 μg/mL (Karthik et al. 2013).

### Brine shrimp lethality of the extracts

**Figure 2.**
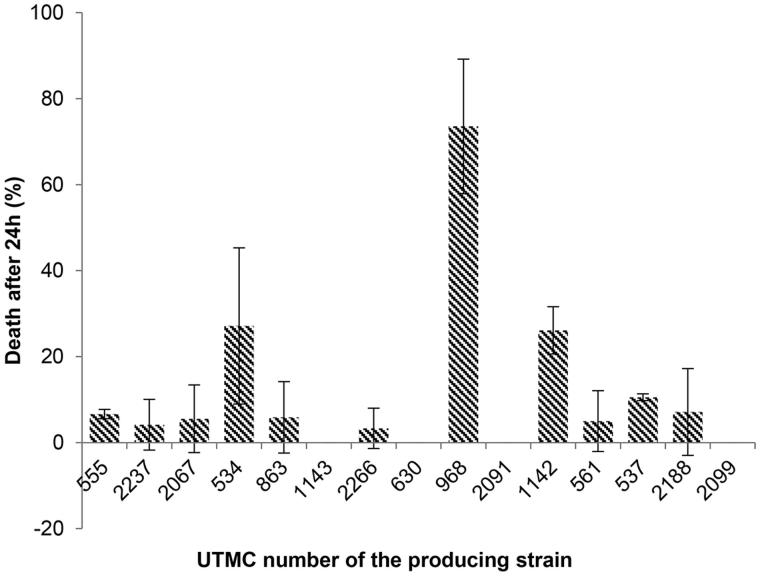
Effect of crude extracts on *Artemia salina* at the concentration of 10 μg/mL after 24 h. The most mortality effect was related to the extract of UTMC 968 and except that no deaths were seen for the extract of UTMC 1143, UTMC 630, UTMC 2091 and UTMC 2099, other samples had more than 3% mortality. Data are mean ± SD of three experiments.

Fifteen out of 52 extracts which presented antioxidant activity were investigated for their primary toxicity effect on *Artemia salina* at concentrations of 10, 5, and 2.5 μg/mL. Only the extract of *Actinokineospora* sp. UTMC 968 had low toxicity (22.7% death) in 2.5 μg/mL while the others did not show any mortality. Potassium dichromate (0.5 mM) led to 100% mortality after 24 h as the positive control while DMSO as the final solving solution did not cause any mortality up to 24 h at the same concentration. The extracts of *Actinokineospora* sp. UTMC 968, *Amycolatopsis* sp. UTMC 534 and *Kribbella* sp. UTMC 1142 showed toxicity up to 74% (LC_50_ 7 μg/mL), 27% (LC_50_ 18.5 μg/mL) and 26% (LC_50_ 19 μg/mL) at 10 μg/mL concentration, respectively (Figure 2). No toxicity was observed in a number of samples including the extract of *Actinophytocola* sp. UTMC 1143, *Nocardia* sp. UTMC 630, *Micromonospora* sp. UTMC 2091 and *Actinomycete* sp. UTMC 2099 while other extracts had less than 10% mortality (LC_50_ >1 mg/mL), that were considered as nontoxic as well. Even though most of the extracts were nontoxic, five extracts were selected for further studies according to their antioxidant potential.

### Superoxide radical scavenging activity

**Figure 3.**
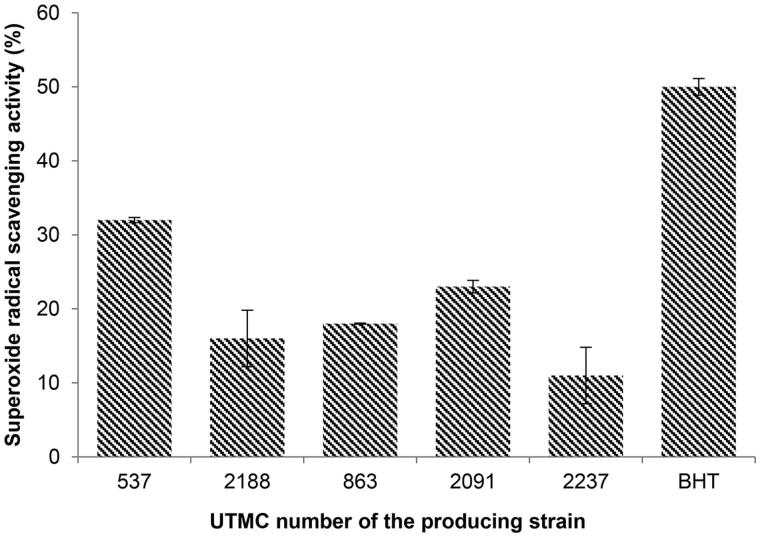
Superoxide radical scavenging activity of five selected extracts at the concentration of 40 μg/mL and BHT at the same concentration. Data are mean ± SD of three experiments.

Five extracts that belonged to different genera of *Actinobacteria* with strong DPPH radical scavenging activity and being nontoxic, were selected for superoxide scavenging activity assessment. The results of the superoxide radical scavenging activity from these strains are presented in Figure 3. The highest scavenging activity was related to the extract of *Saccharothrix ecbatanensis* UTMC 537, which corresponded to 32%. The scavenging activity of BHT at the same concentration was 50%.

The superoxide radical as well as other reactive oxygen species (ROS) contribute to the oxidative damage to living organisms that are involved in many pathological processes such as inflammation, atherosclerosis, cancer, aging, and similar systematic diseases (Halliwell 1992; Cos et al. 1998). In the superoxide radical scavenging assay, the generation of radicals and NBT reduction (Figure 3), was significantly inhibited (32 ± 0.14%) only by the extracts of *Saccharothrix ecbatanensis* UTMC 537 and *Micromonospora echinospora* UTMC 2091, which exhibited 23 ± 0.35 scavenging activity.

### Enzyme inhibitory activity

Five extracts in superoxide anion scavenging activity were investigated for their enzyme inhibitory effect on xanthine oxidase and lipoxygenase enzymes.

#### Xanthine oxidase inhibitory activity

**Figure 4.**
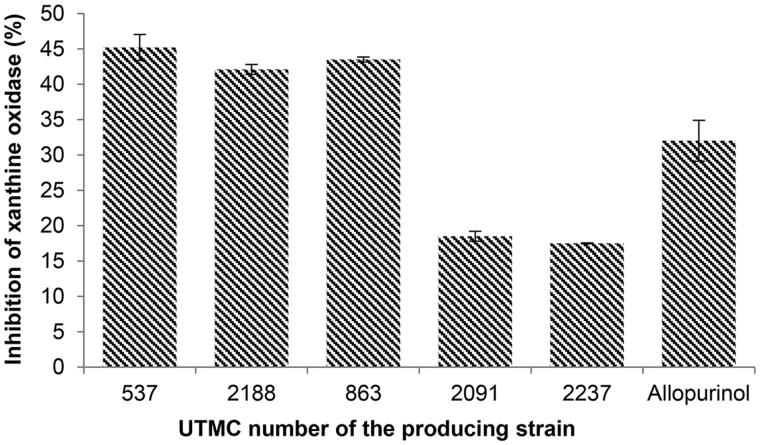
Xanthine oxidase inhibitory activity of selected extracts at the concentration of 12.25 μg/mL and allopurinol at the same concentration. Data are mean ± SD of three experiments.

As seen in Figure 4, three of the antioxidant extracts had higher activity relative to allopurinol (32%) at the concentration of 12.25 μg/mL, amongst them, the most inhibitory activity corresponded to 45.2% which was metabolites produced by *Saccharothrix ecbatanensis* UTMC 537.

The enzyme xanthine oxidase (XO) catalyzes the formation of uric acid from the purines hypoxanthine and xanthine. During this oxidation reaction, superoxide radicals and hydrogen peroxide are produced. In addition, XO is considered as the causing agent of gout. Metabolites produced by *Saccharothrix ecbatanensis* UTMC 537, *Nocardia carnea* UTMC 863, and *Streptosporangium* sp. UTMC 2188 had more XO inhibitory activity in comparison to allopurinol at the same concentration (Figure 4), with more than 40% of enzyme activity inhibition.

#### Lipoxygenase inhibitory activity

**Figure 5.**
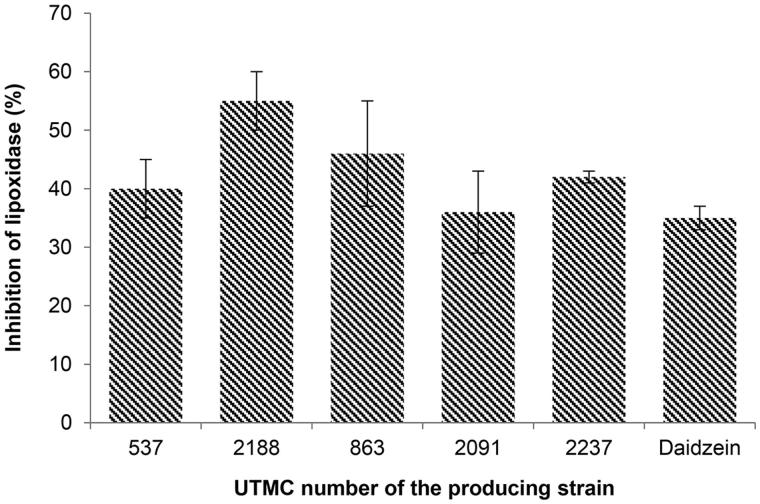
Lipoxygenase inhibitory activity of selected extracts at the concentration of 4 μg/mL and daidzein at the same concentration. Data are mean ± SD of three experiments.

Four out of five antioxidant extracts had more activity compared to daidzein (35% inhibition) at a concentration of 4 μg/mL, as illustrated in Figure 5. The most inhibitory activity was detected by the extract of *Streptosporangium* sp. UTMC 2188, with 55% inhibition of the lipooxygenase activity.

Lipoxygenase is a non-heme iron enzyme that catalyzes the oxidation of polyunsaturated fatty acids as a substrate containing at least one 1*Z*, 4*Z*-pentadiene systems such as linoleic acid, linolenic acid and arachidonic acid in their hydroperoxy products. Recent studies have shown the role of lipoxygenase in some diseases such as cancer and inflammation (Chedea & Jisaka 2011).

### DNA damage inhibition of the antioxidant extracts

**Figure 6.**
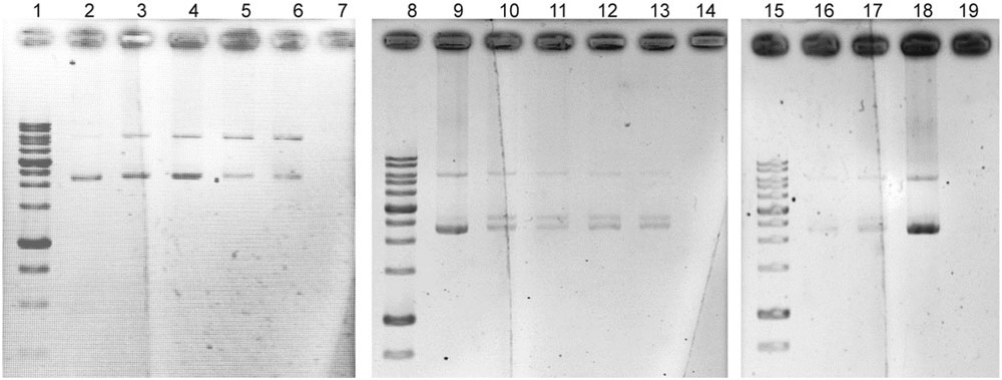
Effects of extracts (1 and 2 mg/mL) against oxidative damage to DNA (pUC19) caused by UV-photolysis of H_2_O_2_ (3%, v/v), lines 2, 9 and 18 are only plasmid DNA (blank: untreated and non-irradiated DNA), lines of 7, 14 and 19 are negative controls (untreated UV-irradiated DNA) in each running set. Line 3 and 4, H_2_O_2_/UV in the presence of UTMC 537 extract with concentrations of 1 and 2 mg/mL, respectively. Line 5 and 6, H_2_O_2_/UV in the presence of UTMC 863 extract at concentrations of 1 and 2 mg/mL, respectively. Line 10 and 11, H_2_O_2_/UV in the presence of UTMC 2188 extract at concentrations of 1 and 2 mg/mL, respectively. Line 12 and 13, H_2_O_2_/UV in the presence of UTMC 2237 extract at concentrations of 1 and 2 mg/mL, respectively. Line 16 and 17, H_2_O_2_/UV in the presence of UTMC 2091 extract at concentrations of 1 and 2 mg/mL, respectively.

The electrophoretic pattern of pUC19 DNA following UV-photolysis of H_2_O_2_ in the absence and presence of five antioxidant extracts at concentrations of 1 and 2 mg/mL are depicted in Figure 6. The faster-moving band represents the native form of supercoiled circular DNA (scDNA) and the slower moving band corresponds to the open circular form (ocDNA). No band was detected in the control line, which implies the entire degradation of the DNA. Partial DNA protection of the extracts especially strain UTMC 537 and strain UTMC 863 was observed as a new intermediate linear DNA (linDNA) band and residues of the scDNA and ocDNA.

The extract of *Streptomyces* sp. VITSTK7 and Streptomyces LK-3 has also reported protecting DNA (pBR322) against UV-induced photolyzed H_2_O_2_-oxidative damage (Thenmozhi and Kannabiran 2012; Karthik et al. 2013). Diazepinomicin produced by a *Micromonospora* sp. has also been able to protect the induced oxidative DNA damage of by H_2_O_2_ in HK-2 cells (Abdelmohsen et al. 2012).

### Anti-hemolytic activity of the antioxidant extracts

**Figure 7.**
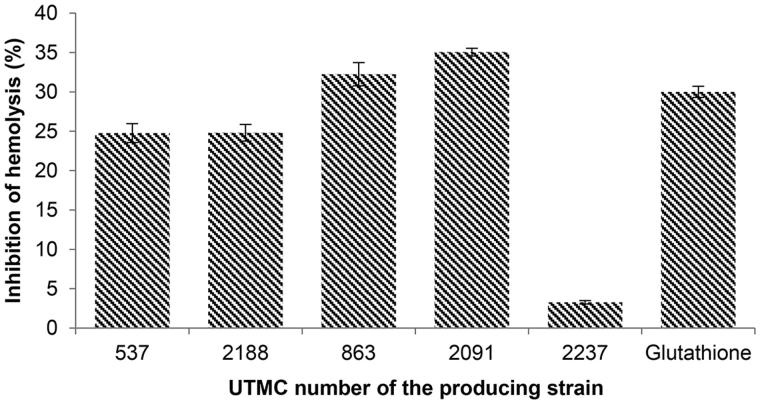
Inhibition of hemolysis (%) induced by 2.5 mM ferrous sulfate with extracts and glutathione as a positive control at the concentration of 100 μg/mL. Data are mean ± SD of three experiments.

The influence of the extracts on the inhibition of erythrocyte hemolysis was observed following incubation of the erythrocytes in the presence of 2.5 mM ferrous sulfate. The extracts provided an average inhibitory effect against erythrocyte hemolysis in the range of 3.3–35%. The extracts of *Nocardia* sp. UTMC 863 (32.2%) and *Micromonospora* sp. UTMC 2091 (35%) had the most inhibitory effect on hemolysis. These two samples had more protective activity compared to glutathione (30% inhibition) at the same concentration of extracts as showed in Figure 7.

The protective effects of kaempferol against ROS-induced hemolysis have been reported up to 87.4% at 100 mg/mL (Liao et al. 2016). The single reported compound with hemolysis inhibition activity from actinobacteria is trehangelins, produced by endophytic *Polymorphospora rubra*. The symmetric structures of trehangelins A (IC_50_ value, 0.1 mg/mL) and C (IC_50_ value, 0.4 mg/mL) have shown inhibitory activity against photo-oxidative hemolysis of red blood cells (Nakashima et al. 2013).

### Cytotoxicity of the selected extracts in the MTT assay

**Figure 8.**
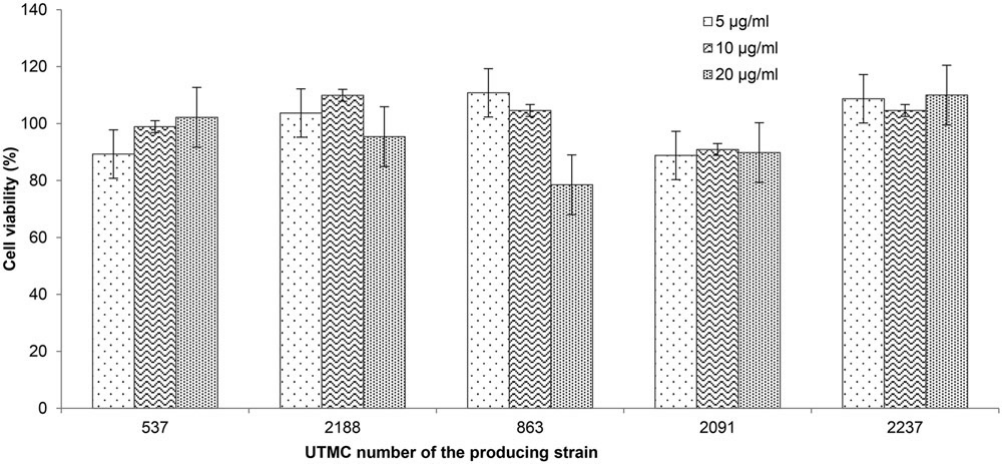
Effect of extracts on human umbilical vein endothelial cells (HUVEC) in MTT assay. Cells treated with 5, 10, and 20 μg/mL of the extracts. Values are presented as mean ± SD.

All of the tested extracts up to 20 μg/mL were not toxic to human umbilical vein endothelial cells (HUVEC) with retaining cell viability higher than 75% compared to the cells treated only with the same amount of DMSO instead of the extracts. As seen in Figure 8, some of the extracts not only did not have any toxic effect; they even had a slightly proliferative effect on the cells.

The human umbilical vein endothelial cells (HUVEC) and fibroblasts are among common normal human cell lines used in the cytotoxicity evaluation of natural extracts. While cell line kept most of their viability in presence of all extracts, in case of four extracts, except extract UTMC 2091, they exhibited proliferative activity on these cells that may indicate their restoration or wound healing activities as well.

### Protection of *Saccharomyces cerevisiae* SOD mutant against antioxidant stress

The osmotic stress of hypertonic growth medium involve oxidative stress by increasing of free radicals especially superoxide (Koziol et al. 2005) and it has reported that the rate of superoxide generation is higher in the CuZnSOD disruptant than in the isogenic wild-type (WT) strain in hypertonic media.

The wild-type yeast grew on all media while the mutant did not grow on media with oxidative stress. Ascorbate and extract of strain *Saccharothrix ecbatanensis* UTMC 537 both could ameliorate growth restriction of sod1 disruptant with rather the same intensity. Therefore, enhanced sensitivity of sod1 mutants to oxidant stress was partially restored in presence of ascorbate and extract of strain UTMC 537 at the concentration of 1 mg/mL.

The genome of *Saccharomyces cerevisiae* has high homology to mammals and yeast models has become one of the bioassay models of pharmaceutical activities. Growth inhibition of sod1 mutant cells caused by osmotic stress was partially restituted by the extract of *Saccharothrix ecbatanensis* UTMC 537. However, the wild-type showed higher growth on extract containing media compared to the negative control and ascorbate containing media which can be attributed to the existence of some micronutrients which has growth promoting effect on wild-type yeast other than its antioxidant activity.

### Secondary metabolite profile of the *Saccharothrix ecbatanensis*

**Figure 9.**
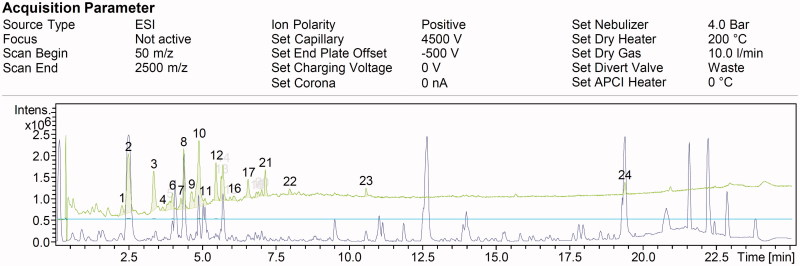
Total ion chromatogram of metabolites detected in crude extract of *S. ecbatanensis* UTMC 537 (Y: Intensity and X: Retention time). Metabolites with new structure were detected as compound No.13 in 5.65 min RT with 387.1116 [M + H]+ and compound No. 20 in 7.03 min RT with 623.2030 [M + H]+.

Analysis of produced secondary metabolites by *S. ecbatanensis* in ISP2 medium revealed 21 dominant compounds and some more other compounds produced in minor amounts (Figure 9). The strain *S. ecbatanensis* showed the ability to produce two new metabolites in the dereplication process conducted based on the database search of the high resolution molecular weights of the detected metabolites obtained from its spectrum. The probable new compounds with molecular weight values of 386.1116 *m/z* (RT 5.65) and 622.2030 *m/z* (RT 7.03) had the maximum UV absorption at 218/316 nm and 218 nm, respectively. The respected molecular weights could not be matched with the compounds in the Dictionary of Natural Products database and SciFinder. The compound with 387.1116 [M + H]+ molecular weight and C_20_H_19_O_8_ showed 18% scavenging activity of DPPH radicals.

It can be concluded that some of the antioxidant activities revealed for the extract of *S. ecbatanensis* can be attributed to the compound with 386.116 D. However, some of these antioxidant activities can also be originated from other known molecular weights in the spectra which are already described compounds.

Due to the insufficient amount of the retrievable compound, its structure could not be elucidated. In order to obtain sufficient amount of the compounds for structural determination and further bioassay analysis, the strain must be subjected to an extensive optimization process, large-scale production/purification process or genetic manipulation of the strain to induce its higher amount of production.

## Conclusions

There are numerous studies on the importance of antioxidants in the prevention of chronic diseases such as cancer and coronary heart disease (Rao and Agarwal 1999; Scalbert et al. 2005). Additionally, the probable application of antioxidants in the treatment or prevention of several diseases such as Parkinson (Offen et al. 1996) and diabetes (Scalbert et al. 2005) has been recognized.

In the present study, extracts of 52 rare Actinobacterial strains from 11 genera were screened for their antioxidant properties, while in other studies mainly the capability of members belong to *Streptomyces* genus has been investigated. Fifteen extracts were markedly able to reduce the free radical 2,2-diphenyl-1-picrylhydrazyl (DPPH) to the colored non-radical (Figure 1) form of diphenylpycrilhydrazine that is based on the reduction in the presence of a hydrogen donating agent (Brand-Williams et al. 1995). Toxicity screening of 15 extracts was performed using the brine shrimp lethality assay (Figure 2) that is a trustworthy and inexpensive bioassay for primary evaluation of toxicity in drug discovery studies (Meyer et al. 1982; Carballo et al. 2002). Based on the results of these two preliminary screenings, five nontoxic extracts with the most potent DPPH radical scavenging activity (more than 25%) were evaluated for the scavenging of other radicals, and additionally for enzyme inhibitory, anti-hemolytic and cytotoxicity activity.

It can be inferred that these five extracts and especially strain UTMC 537, with high ability in the scavenging of superoxide radicals and inhibition of xanthine oxidase and Lipoxygenase, probably produces components for reducing oxidative damage related to these enzymes and their radical products. Therefore, the active metabolites in the extract of *Saccharothrix ecbatanensis* UTMC 537 (DSM 45486) might be considered as a potential antioxidant, anti-inflammation, anti-hemolytic and anti-gout agent. The crude extract of this strain maintained the viability of the eukaryotic cell models including *Artemia salina* and human umbilical vein endothelial cells up to 90% at the concentration of 20 µg/mL which showed the absence of compounds with cell toxicity among its secondary metabolites. This extract not only did not show a hemolytic effect on human blood cells, but also could inhibit hemolysis induced by oxidant induction.

Due to the increasing interest in finding natural antioxidants with higher capacity and lower side effects, in recent studies, there are a number of reports on the antioxidant activity of microbially produced metabolites. However, the antioxidant activity of non*-Streptomyces* members is rarely investigated. The antioxidant activity of methanolic extract of an endophytic *Streptomyces* sp. is reported in DPPH scavenging and superoxide anion radical scavenging assay (Christhudas et al. 2013). The DPPH radical scavenging activity of a marine *Streptomyces* sp. (Karthik et al. 2013) is also reported. The compound 5-(2,4-dimethylbenzyl) pyrrolidin-2-one (DMBPO) from a *Streptomyces* strain was exhibited DPPH scavenging activity with 44.1% inhibition at the concentration of 5 μg/mL (Saurav and Kannabiran 2012). In another study, four compounds, 2,3-dihydroxybenzoic acid, 2,3-dihydroxybenzamide, *N*-carbamoyl-2,3-dihydroxybenzamide and 2-acetamido-3-(2,3-dihydroxybenzoylthio) propanoic acid were isolated from a marine-derived actinobacterium which exhibited DPPH radical scavenging activity with ED_50_ value of 10.3, 14.6, 14.4, and 13.0 μM, respectively, in comparison with the BHT (34.7 μM) (Sugiyama and Hirota 2009). The compound dermacozine C from the rare actinobacterium, *Dermacoccus* sp. MT1.2 exhibited DPPH radical scavenging with an IC_50_ value of 8.4 µM against ascorbate with an IC_50_ value of 12.1 (Abdel-Mageed et al. 2010).

In conclusion, all five prominent extracts were found to possess multiple types of antioxidant activities, including scavenging of different free radicals, enzyme inhibitory and anti-hemolytic activity. Additionally, none of them contained cytotoxic compounds. Therefore, all of these five extracts could comprise valuable multifunctional antioxidant compounds which can be exploited in future analysis. This multifunctional antioxidant activity is of distinctive magnitude due to the numerous antioxidant stress mechanisms that occur in biosystems.

Since there was no obvious correlation between enzyme inhibition and radical scavenging activity among the extracts in this study, it can be concluded these activities originate from irrelevant mechanisms. However, the presence of the compounds that prevent the formation of the ROS in addition to their scavenge in the case of generation, can designate the *Saccharothrix ecbatanensis* UTMC 537 as a precious source for the discovery of multipotent antioxidant compounds.

Whereas this study was performed using crude extracts, their effective components in the pure condition are anticipated to have significant antioxidant activity compared to the positive controls, especially, *Saccharothrix ecbatanensis* UTMC 537, which was the most multi-mechanism antioxidant producing and a new species in the genus *Saccharothrix* as well (Mohammadipanah et al. 2015). In spite of no report of the antioxidant secondary metabolite from this genus, the inter-species differences of genomes manifest considerable secondary metabolite diversity that can lead to structurally new compounds with antioxidant activity in this new species of *Saccharothrix*.
